# Religiosity, Meaning-Making and the Fear of COVID-19 Affecting Well-Being Among Late Adolescents in Poland: A Moderated Mediation Model

**DOI:** 10.1007/s10943-021-01375-7

**Published:** 2021-08-21

**Authors:** Dariusz Krok, Beata Zarzycka, Ewa Telka

**Affiliations:** 1 Institute of Psychology, University of Opole, Plac Staszica 1, 45-052 Opole, Poland; 2 Institute of Psychology, John Paul II Catholic University of Lublin, Lublin, Poland; 3 Department of Radiotherapy, Maria Sklodowska-Curie National Research Institute of Oncology, Gliwice, Poland

**Keywords:** Religiosity, Meaning-making, Fear of COVID-19, Subjective well-being, Late adolescents

## Abstract

Adolescents have come to be greatly affected by the COVID-19 pandemic and the ensuing containment measures in recent months. The aim of the present study was to examine the relations among religiosity, meaning-making, fear of COVID-19, and subjective well-being within a moderated mediation model. Three hundred and sixteen late adolescents (173 women and 143 men) in Poland volunteered to take part in the study. The results show that meaning-making mediated relationships between religiosity and life satisfaction, religiosity and positive affect, and religiosity and negative affect. In addition, these mediation effects were moderated by the fear of COVID-19. Specifically, the indirect effects were stronger for adolescents with high fear than for those with low fear, which indicates that fear of COVID-19 serves as a ‘warning’ factor.

## Introduction

In 2020, the COVID-19 pandemic paralysed the world and dramatically disrupted public life. By the end of summer 2020, over one million people had died globally from the coronavirus disease and the number of infected people exceeded 34 million (Johns Hopkins University, 2020); health services in many countries were pushed to the limits of their capacity to help the sick. The pandemic generated high levels of fear and anxiety among people and led to significant changes in their lifestyles, including religious beliefs and behaviour (Lucchetti et al., 2020). This also became relevant to young people, who although initially not heavily affected by the disease, began to experience a range of negative mental health consequences by the end of summer 2020 (Cunningham et al., 2020). Even though the majority of older adolescents did not require hospital treatment, they still experienced adverse effects in terms of subjective well-being. It is thus important to examine to what extent fear of COVID-19 influences relationships among religiosity, meaning-making and subjective well-being (SWB) in late adolescents.

### Relationships Between Religiosity and Subjective Well-Being

Although empirical evidence clearly points to positive relationships between religiosity and SWB (Hardy et al., 2019; Villani et al., 2019), some important issues regarding potential mediating factors specific to late adolescents still remain unexplored and require more thorough investigation. In addition, the complexity of associations between religiosity and SWB indicates the presence of interrelated mediation and moderation mechanisms.

Research has shown that the presence of meaning in life mediates the relationship between religiosity and life satisfaction (i.e. the cognitive dimension of SWB), but not between religiosity and positive and negative affect (i.e. the affective dimension of SWB). However, search for meaning in life did not play any mediational role in these relationships (Krok, 2014). One of the dimensions of sense of coherence, i.e. meaningfulness, was found to mediate the association of spirituality with life satisfaction in older adults (Cowlishaw et al., 2013). Inverse associations were also found between spiritual and religious beliefs and depression (a factor which is considered a negative indicator of SWB), as well between sense of coherence and depression in Greek adults (Anyfantakis et al., 2015). These results suggest that meaning-based appraisals of life events may influence the way in which a religious/spiritual motivation is related to well-being.

### Meaning-Making as a Mediator

There have been studies demonstrating the mediating role of meaning-making between specific measures of religiosity and well-being. Meaning-making can be conceptualised as one’s cognitive process aimed at perceiving and understanding life events in a different way and assimilating them within the consistent structures of personal beliefs and goals. It represents the ability to deliberately make meaning of ongoing events and situations (Park, 2013; Van den Heuvel et al., 2009). Meaning-making is thus a dynamic process that involves one’s conscious reflection and interpretation. Examining a Polish adult sample, Zarzycka et al. (2020) revealed that meaning-making was a mediator in the association between religious struggles and psychological well-being. In a sample of Christian adults who experienced a loss of a loved one, Lichtenthal et al. (2011) showed that challenges with meaning-making partially explained the relationship of negative religious coping with prolonged grief.

Meaning-making coping (i.e. a form of meaning-making focussed on coping with difficult events) also mediated the relationship of intrinsic religion with SWB in bereaved individuals (Park, 2005) and the effect of intrinsic religion on quality of life in people with schizophrenia (Tabak & de Mamani, 2014). Although those studies applied a variety of measures examining the associations between religiosity, meaning-making and well-being, to date, there has not been research to directly investigate meaning-making as a mediator between religiosity and SWB in a group of late adolescents. Potential changes in religiosity, which are characteristic of young people, are very likely to initiate new modes of thinking about meaning and purpose that may affect their well-being.

### The Explanatory Context of the Meaning-Making Model

The associations between religiosity, meaning-making and well-being can be more deeply understood within the context of the Meaning-Making Model, which provides a constructive theoretical background to our analyses (Park, 2010; Park & George, 2013). The model proposes that people are ‘equipped’ with motivational resources and cognitive orienting systems that allow them to analyse and interpret daily experiences. When encountering a demanding or stressful situation, people appraise the situation and assign meaning to it. The discrepancy between people’s global beliefs and goals and situational meanings causes distress, which in turn initiates meaning-making, cognitive attempts to restore coherence between specific aspects of global and situational meaning. Religion is often a significant part of meaning-making processes for at least two reasons: (1) for many people, religious beliefs and goals are deeply embedded in their global system of meanings, and (2) most religions offer ways of coping with difficulties and suffering, which enable people to find meaning and purpose (Park, 2013).

Empirical research has demonstrated the usefulness of the Meaning-Making Model in investigating relationships between religiosity and well-being. Examining young people who had experienced a serious traumatic event within the framework of the model, Park and Gutierrez (2013) found that personal beliefs in God were positively associated with happiness but not with life satisfaction. The relationship between the religious meaning system and psychological well-being was also mediated through two coping strategies: problem-focussed and meaning-focussed in older cancer patients, which was thus interpreted from a meaning-making perspective (Krok et al., 2019). Taking into account that the stage of late adolescence is connected with substantial shifts in religious beliefs and behaviour and searching for life goals, young people’s well-being can be particularly sensitive to any changes and reinterpretations in their global meaning.

### The Moderation Effect of Fear of COVID-19

In view of the high transmissibility of the coronavirus disease and its health hazards, it is not surprising that fear of COVID-19 may strongly influence individuals’ well-being. Furthermore, recent research has revealed that fear of COVID-19 affects mental health, well-being and behaviour in adolescents (Masuyama et al., 2020; Oosterhoff et al., 2020). Due to social distancing and self-isolation regulations implemented by many countries around the world, young people witness dramatic changes in their environment and, consequently, experience a wide range of negative mental health symptoms (e.g. anxiety, fear, sadness, feelings of frustration and worry, impulsivity, and difficulty concentrating). They may have far-reaching consequences on later behavioural profiles among young people.

There is also empirical evidence indicating that fear plays a moderation role in regard to factors closely related to well-being. Examining work-related experiences during the coronavirus disease, Gasparro et al. (2020) found that the fear of COVID-19 was a moderator of the relationship between perceived job insecurity and depressive symptoms. Fear of COVID-19 also acted as a moderator of the indirect effect of sense of coherence in the relationship between stress and life satisfaction in an adult sample (Dymecka et al., 2020). Fear of COVID-19 played a buffering role in this relationship as it weakened the effect of stress on sense of coherence. In addition, religious beliefs can be conducive to reducing fear and anxiety as they provide a sense of security (Exline et al., 2014), explain existential questions (Park, 2013), and buffer stress through effective ways of coping (Lichtenthal et al., 2011). However, no previous studies have explored the potential moderation effect of fear of COVID-19 in associations among religiosity, meaning-making and SWB in late adolescents. Given that fear was reported to affect meaning-making processes by prompting people to reappraise their current situation and make efforts to find meaning and purpose (Ownsworth & Nash, 2015; Thomsen et al., 2016), it is likely that fear of COVID-19 interacts with meaning-making in relation to SWB.

This assumption finds support in the Meaning-Making Model which posits that difficult and stressful situations can activate processes of meaning-making, through which people make attempts to reduce discrepancies between the appraised meanings of a demanding event and their global beliefs and goals (Park & Blake, 2020). Although research showed that meaning-making was related with higher well-being and better adjustment, especially if satisfactory and beneficial meanings were formed (Park & George, 2013), it is still unknown how fear of COVID-19 would interact with meaning-making in regard to the cognitive and affective dimensions of SWB.

### The Present Study

**Fig. 1:**
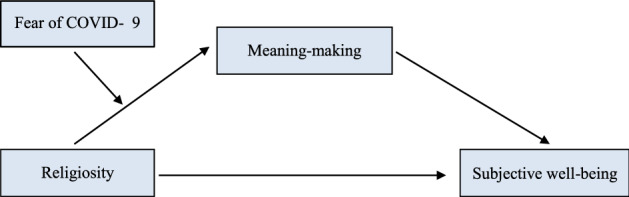
The general moderated mediation model

The present study aims to examine a moderated mediation model to deeply understand the relationship between religiosity with subjective well-being among late adolescents (Fig. 1). Based on the Meaning-Making Model and past research, we formulated three hypotheses: (1) Meaning-making will mediate the relationship between religiosity and the dimensions of subjective well-being; higher religiosity is associated with stronger meaning-making, which then relates to greater subjective well-being. (2) Fear of COVID-19 moderates the indirect effect between religiosity and life satisfaction and positive affect (positive indicators of subjective well-being) through meaning-making; the indirect effect is stronger when fear is high vs. low. (3) Fear of COVID-19 moderates the indirect effect between religiosity and negative affect (negative indicator of subjective well-being) through meaning-making; the indirect effect is weaker when fear is high vs. low.

To establish an adequate sample size N, we conducted a priori power analysis G * as recommended by Faul et al. (2009); it was computed as a function of power level (1-*β*), pre-specified significance level *α* = 0.05, and test power (1-*β*) = 0.90. The required sample size of *N *= 280 was estimated as sufficient for our examination. The sample is then considered to be representative for late adolescents in our country.

## Method

### Participants and Procedure

The participants were three hundred and sixteen late adolescents (173 women and 143 men) who volunteered to take part in our study. They ranged in age from 17 to 24 years (*M* = 21.58, SD = 2.03). To guarantee the representativeness of the sample, quota sampling was put on the following characteristics: gender, age, and types of education vs. work. Due to the sociodemographic factors in the surveyed country, participants were predominantly White and Christian, with a small percentage of atheists/agnostics. This social structure of the respondents can be attributed to specific historical and cultural factors of the society in which our study was conducted (i.e. Poland). Participant demographic characteristics are given in Table 1.

**Table 1:** Participant demographic characteristics

Participants	M	SD
Age	21.58	2.03
	*N*	*%*
Gender		
Male	143	45.3
Female	173	54.7
Study vs. work status		
Basic vocational education	44	13.9
High school education	116	36.7
University education	89	28.2
Full-time work	67	21.2
Religion		
Catholic	262	82.9
Protestant	22	7.0
Atheist	14	4.4
Agnostic	18	5.7
Personal knowledge of people who had been ill with COVID-19		
Yes	249	78.8
No	67	21.2
Having had laboratory-confirmed COVID-19		
Yes	10	3.2
No	206	96.8

Participants were recruited in schools, colleges, and through social, religious and cultural organisations. They were given questionnaires in person or were sent them with necessary information and explanations. Four individuals who had initially agreed to participate declined at the beginning of the study; the following reasons were given: difficulties in understanding some items and negative attitudes towards religion. Once the questionnaires were completed, the participants returned them to the research assistants. The study was anonymous and participants could opt out at any time. The study protocol was approved by the University Ethics Committee, and informed consent was obtained from participants (adults) or their parents (minors). After the study, those participants who had any further questions (e.g. related to the aim of the study, availability of the results, or psychological aspects of the COVID-19 pandemic) were able to contact the research assistants.

### Measures

#### Religiosity

The Religious Meaning System Questionnaire (RMS) was used to measure a level of religiosity (Krok, 2014). The RMS consists of twenty items rated on a seven-point Likert scale, ranging from 1 (very strongly disagree) to 7 (very strongly agree). The questionnaire measures religiosity conceptualised in terms of the religious meaning system that enables individuals to perceive and interpret their life through the lens of significance and purpose (Krok, 2014). The sample items are: ‘Thinking about my life, I take into account religious norms’ or ‘I think that religion helps me in finding purpose in life’. A high score represents a higher level of religiosity. The Cronbach’s α for the current study was 0.89.

#### Life Satisfaction

The Satisfaction with Life Scale (SWLS; Diener et al., 1985) was applied to evaluate global cognitive judgments of subjective well-being. It is a popular and well-validated scale that represents the degree to which people are satisfied with their lives as a whole. The SWLS comprises five items rated on a five-point Likert scale, ranging from 1 (absolutely untrue) to 5 (absolutely true). A higher score indicates greater satisfaction with life. The Cronbach’s α for the present study was 0.86.

#### Positive and Negative Affect

The Positive and Negative Affect Schedule (PANAS-X; Watson & Clark, 1991) measures two broad dimensions of emotional experience, i.e. positive affect and negative affect, as well as 11 specific affective emotional states that emerge from within these general dimensions. It is used to assess the affective component of subjective well-being. The PANAS-X includes 60 items rated on a five-point scale, from 1 (very slightly or not at all) to 5 (extremely). Due to the aim of the current study, we only used the positive and negative affect subscales. As we aimed at estimating a relatively stable level of affect, the time instruction was: ‘to what extent you have felt this way during the past two weeks’. The Cronbach’s α for the present study was 0.86 for positive affect and 0.87 for negative affect.

#### Meaning-Making

The Meaning-Making Questionnaire (MMQ, Krok & Zarzycka, 2020) was used to measure individuals’ cognitive capacity to comprehend and assimilate challenging or ambiguous life events into coherent structures of personal meaning, beliefs and goals. It comprises eight items rated on a five-point Likert scale, ranging from 1 (never) to 5 (very often). A higher score represents a stronger process of meaning-making. The sample items are: ‘I try to discover what is most important in a given situation’ or ‘I focus on meaning and purpose in current events’. The Cronbach’s α for the present study was 0.87.

#### Fear of COVID-19

The level of fear experienced by people during the coronavirus pandemic was evaluated by the Fear of COVID-19 Scale (Dymecka, 2020). It contains six items rated on a five-point Likert scale, from 1 (strongly disagree) to 5 (strongly agree). The scale measures emotional experiences of anxiety and apprehension triggered by the current pandemic. As we aimed at estimating a relatively stable level of fear of COVID-19, the time instruction was: ‘to what extent you have felt this way during the past two weeks’. The sample items are: ‘I fear an extended hospital stay in case of being infected by coronavirus’, or ‘I am scared of losing my life due to coronavirus infection’. The Cronbach’s α for the present study was 0.83.

### Statistical Methods

The present study was based on a cross-sectional design. All analyses were conducted in SPSS Statistics 21. First, we calculated descriptive statistics and correlational analyses to establish relationships among religiosity, subjective well-being, meaning-making and fear of COVID-19 among late adolescents. Second, mediation analysis using Model 4 with one mediator was performed with bootstrap procedures suggested by Hayes (2018) (95% bias-corrected confidence intervals, 5000 bootstrap resamples); the aim was to examine the mediating effect of meaning-making in relationships between religiosity and the dimensions of subjective well-being (life satisfaction, positive affect, negative affect). Third, moderation mediation analysis was carried out to examine whether fear of COVID-19 was a moderator of the indirect effects between religiosity and subjective well-being through meaning-making (Model 7). Statistical significance was determined with bootstrap procedures (95% bias-corrected confidence intervals, 5000 bootstrap resamples) (Hayes, 2018).

## Results

### Preliminary Analyses

First, descriptive statistics and correlations among study variables were calculated (Table 2); most of the bivariate associations turned out to be statistically significant.

**Table 2:** Means, standard deviations, and correlations among religiosity, subjective well-being, fear of COVID-19, and meaning-making

Variables	*M*	*SD*	1	2	3	4	5	6	7
1. Age	21.58	2.06	‒						
2. Religiosity	4.13	1.74	– 0.05	‒					
3. Life satisfaction	3.98	1.22	– 0.10	0.21^***^	‒				
4. Positive affect	3.09	0.81	– 0.02	0.12*	0.58^***^	‒			
5. Negative affect	2.25	0.89	0.01	– 0.01	– 0.13*	– 0.14^**^	‒		
6. Meaning-making	3.61	0.87	– 0.07	0.32^***^	0.31^**^	0.34^**^*	– 0.29^***^	‒	
7. Fear of COVID-19	3.42	0.97	– 0.13*	0.18^**^	0.09	0.02	0.13*	0.13*	‒

*p < .05; ** *p* < .01; *** *p* < .001

Age was negatively related to only one variable—fear of COVID-19. As expected, religiosity was positively associated with life satisfaction, positive affect, and meaning-making. Surprisingly, however, religiosity was also positively associated with fear of COVID-19. Meaning-making positively correlated with life satisfaction and positive affect, but negatively correlated with negative affect. Regarding fear of COVID-19, this factor was positively associated with negative affect, and, rather surprisingly, with meaning-making. There were no statistically significant relations between fear of COVID-19 and positive indicators of subjective well-being, i.e. life satisfaction and positive affect. Within the internal structure of subjective well-being, life satisfaction (the cognitive dimension) was positively associated with positive affect and negatively associated with negative affect (the affective dimension). Positive affect was also negatively correlated with negative affect.

### Mediation Analysis

Mediation analysis was conducted to examine whether meaning-making would mediate the relationships between religiosity and three dimensions of subjective well-being, i.e. life satisfaction, positive affect and negative affect, respectively (Table 3).

**Table 3:** Mediation estimates for meaning-making in the relationship of religiosity with subjective well-being

Variables	B	SE	*t*	Model *R*^2^
Direct effects				
Religiosity—Meaning-making	0.22	0.04	5.89***	0.10***
Meaning-making—Life satisfaction	0.38	0.08	4.80***	
Religiosity—Life satisfaction	0.12	0.06	2.13*	0.11***
Meaning-making—Positive affect	0.31	0.05	6.06***	
Religiosity—Positive affect	0.01	0.03	0.20	0.12***
Meaning-making—Negative affect	– 0.33	0.06	– 5.64***	
Religiosity—Negative affect	0.07	0.04	1.61	0.09***

	Effect	SE	LLCI	ULCI
Indirect effects				
Religiosity—Meaning-making—Life satisfaction	0.08	0.02	0.01	0.23
Religiosity—Meaning-making—Positive affect	0.07	0.02	0.04	0.11
Religiosity—Meaning-making—Negative affect	− 0.08	0.02	− 0.11	− 0.05

*p < .05, ***p*p* < .001

The indirect effects demonstrated that meaning-making mediated the relationships between religiosity and life satisfaction, religiosity and positive affect, and religiosity and negative affect. However, the directions of the relationships varied between the specific variables. Religiosity related to higher meaning-making, which in turn was related to a higher level of life satisfaction and positive affect. In contrast, religiosity related to higher meaning-making which in turn was related to a lower level of positive affect. Hypothesis 1 was therefore fully confirmed.

### Moderated Mediation Analysis

Hypotheses 2 and 3 assumed that the mediation process described above would be moderated by fear of COVID-19. Therefore, a set of moderated mediation analyses was performed to verify those assumptions (Table 4).

**Table 4:** Moderated mediation estimates for subjective well-being outcomes

Variables	B	SE	t	Model R^2^
Direct effects				
Religiosity—Meaning-making	0.21	0.04	5.62***	
Fear of COVID-19—Meaning-making	0.06	0.04	1.28	0.12***
Meaning-making—Life satisfaction	0.38	0.08	4.80***	
Religiosity—Life satisfaction	0.12	0.05	2.13*	0.11***
Meaning-making—Positive affect	0.31	0.05	6.06***	
Religiosity—Positive affect	0.01	0.03	0.21	0.12***
Meaning-making—Negative affect	− 0.33	0.06	– 5.65***	
Religiosity—Negative affect	0.07	0.04	1.61	0.09***
Interaction: Religiosity x Fear of COVID-19	0.08	0.04	2.01*	

	Effect	SE	LLCI	ULCI
*Conditional indirect effects*				
Life satisfaction as a dependent variable				
Low fear of COVID-19 × Meaning-making	0.05	0.03	0.01	0.11
Moderate fear of COVID-19 × Meaning-making	0.08	0.02	0.04	0.13
High fear of COVID-19 × Meaning-making	0.11	0.03	0.06	0.18
Positive affect as a dependent variable				
Low fear of COVID-19 × Meaning-making	0.04	0.02	0.01	0.09
Moderate fear of COVID-19 × Meaning-making	0.07	0.02	0.04	0.11
High fear of COVID-19 × Meaning-making	0.09	0.02	0.05	0.14
Negative affect as a dependent variable				
Low fear of COVID-19 × Meaning-making	– 0.05	0.02	– 0.09	– 0.01
Moderate fear of COVID-19 × Meaning-making	– 0.07	0.02	– 0.11	– 0.04
High fear of COVID-19 × Meaning-making	– 0.10	0.03	– 0.15	– 0.05
Index of moderated mediation				
Life satisfaction as DV	0.03	0.02	0.01	0.07
Positive affect as DV	0.03	0.01	0.01	0.06
Negative affect as DV	– 0.03	0.01	– 0.06	– 0.01

***p < .001; **p < .01; *p < .05; *DV* dependent variable

The results showed significant directs effect between religiosity and meaning-making (positive direction), meaning-making and life satisfaction (positive direction), religiosity and life satisfaction (positive direction), meaning-making and positive affect (positive direction), and meaning-making and negative affect (negative direction). In addition, the interaction between religiosity and fear of COVID-19 was significant for meaning-making.

Conditional indirect effects were calculated for three consecutive indicators of subjective well-being: life satisfaction, positive affect and negative affect. The results show that all the three indices of moderated mediation achieved a level of statistical significance (Table 4). This indicates that fear of COVID-19 was a moderator of the indirect effects of religiosity to life satisfaction, positive affect, and negative affect, respectively, through meaning-making. Specifically, for life satisfaction and positive affect, the conditional indirect effect for late adolescents with high fear of COVID-19 was stronger than for late adolescents with low fear; in these cases, the sign of the conditional indirect effects was positive. A slightly different situation occurred in the case of negative affect in which, despite the conditional indirect effect being stronger for adolescents with a high fear of COVID-19 than for adolescents with low fear, the sign of the effect was negative (see Table 4). These results thus fully support hypothesis 2 and partially support hypothesis 3.

## Discussion

The aim of the present study was to verify whether fear of COVID-19 moderated the mediating relationship between religiosity and SWB through meaning-making in late adolescents. Our findings support both the mediating and moderated mediation effects that clearly demonstrated a complex pattern of associations among these variables. To our knowledge, this is the first study examining interactive effects among religiosity, meaning-making, fear of COVID-19 and SWB among Polish late adolescents during the COVID-19 outbreak.

### The Mediating Function of Meaning-Making

Hypothesis 1 was fully confirmed as the results showed that meaning-making mediated the relationship between religiosity and all the three indicators of SWB: life satisfaction, positive affect and negative affect. Specifically, higher religiosity was related to stronger meaning-making, which in turn related to a higher level of life satisfaction and a lower level of negative affect. These results are compatible with previous studies which pointed out a significant role of meaning-making in shaping relationships between religion and well-being (Krok, 2014; Park, 2013; Tabak & de Mamani, 2014). Meaning-making processes seem to be particularly important for young people during the COVID-19 pandemic, mainly due to their adaptive character. Containment measures (i.e. school closures, online learning, social distancing) have drastically altered young people’s everyday life and considerably affected their mental health and quality of life (Schlack et al., 2020; Sham et al., 2020). This situation has placed particular demands on comprehending the world from a new perspective and reorganising their important beliefs and goals, including those which belong to the religious realm. Due to its ability to restore coherence among conflicting aspects of global and situational meanings, meaning-making enables late adolescents to appraise stressful events in a more positive religious light (situational meaning) and modify their religious/spiritual beliefs and values so that they do not conflict with current experiences (global meaning). This interpretation finds support in the Meaning-Making Model, which assumes that in times of personal crisis, individuals tend to reduce their sense of uncertainty and restore a vision of current events and their own life in order to make them coherent and comprehensible (Park & George, 2013).

### Moderating Effects of Fear of COVID-19 for Life Satisfaction and Positive Affect

The main results of the present study were obtained within the moderated mediation effects. Hypothesis 2 was confirmed as the moderated mediation effect of fear of COVID-19 turned out to be significant for life satisfaction and positive affect with the indirect effect being stronger for adolescents characterised by high fear than for those with low fear. This finding confirms previous studies conducted on adult (Thomsen et al., 2016) and clinical (Ownsworth & Nash, 2015) samples in which fear intensified meaning-making processes by motivating individuals to more actively engage in reappraising their current situation and finding purpose, which in turn led to better outcomes in well-being.

However, our study extends previous research on at least two points. First, it demonstrates that fear of COVID-19 is also a vital ‘protective’ factor for young people who, although they had not paid too much attention to the threats posed by coronavirus in the early stages of the pandemic, eventually came to realise its significance and potential consequences through meaning-making processes. This is partially understandable if we take into account the higher than average level of anxiety experienced by young people. Second, our study specifies that fear of COVID-19 intensifies the effect of religiosity on meaning-making, and religiosity consequently fulfils its positive function in increasing life satisfaction and positive affect. In other words, fear of COVID-19, which reflects anxiety and worry about the danger of the coronavirus pandemic, makes it easy for young people to use religious resources. In addition, fear of COVID-19 can prompt late adolescents to use specific religious resources, for example, those which are based on meaning or values (Krok et al., 2019; Park, 2013). They are likely to enable individuals to understand the situation from a different perspective and provide ultimate motivation.

This finding is particularly interesting as it shows not only that fear motivates meaning-making, as demonstrated by past studies (Ownsworth & Nash, 2015; Thomsen et al., 2016), but also that the fear can facilitate the use of religious resources by young people during the pandemic. First, given the presence in many religions of appeals and norms encouraging care and protection of health, fear of the potential dangers associated with the COVID-19 pandemic can prompt young people to observe safety rules more carefully. Second, the risk of a coronavirus infection and the resulting distress can make young people turn to religious activities (e.g. prayer, religious services) for solace and support. These religious activities can be regarded as religious coping strategies that aim at alleviating the stress and discomfort caused by fear of COVID-19 (Counted et al., 2020). In this sense, fear can be an adaptive form of emotional response directed towards survival and optimal functioning in the face of life-threatening circumstances.

One potential explanation may be that fear of COVID-19 could play a positive role in influencing life satisfaction and positive emotions among late adolescents by activating meaning-making processes, which provide the individuals with danger signals that warn of potentially high risks of infection and harmful health consequences (Kanekar & Sharma, 2020; Park & Blake, 2020). In the context of health-related behaviour, awareness of religious beliefs and norms related to taking care of one’s own health and responsibility for the health of others can act as a ‘booster’ for using positive religious coping strategies, as so doing could lead to achieving higher well-being. Religiously related fear can motivate young people’s adaptive behaviour by prompting them to appraise the pandemic situation in terms of threat, loss or challenge, and reformulate their previous views on the pandemic through a religious lens (Boss et al., 2015; Schachter & Ben Hur, 2019). Fearing the negative outcomes posed by COVID may thus trigger cognitive activities that enable adolescents to see or understand the pandemic situation in a different way and to revise their convictions in order to restore internal consistency.

### Moderating Effects of Fear of COVID-19 for Negative Affect

Hypothesis 3 was only partially confirmed. The moderated mediation effect of fear of COVID-19 turned out to be significant for negative affect; yet, contrary to our expectations, the indirect effect was stronger for adolescents with high fear than for those with low fear. This result is an interesting case as it sheds new light on the role played by fear of COVID-19 in arousing negative feelings in the context of well-being (Schlack et al., 2020). Although fear of COVID-19 was positively related to negative affect, the sign of the moderated mediation effect (i.e. fear of COVID-19 × meaning-making) was negative. Thus, we can conclude that at high levels of fear, religiosity was associated with lower negative affect than at moderate or low levels of fear. The comparison of the moderating effects for positive and negative affect showed that although their values were almost equal, they had different directions. Furthermore, for positive affect, fear was positively related to meaning-making, while for negative affect, fear was negatively related to meaning-making.

Considering that lower negative affect indicates higher SWB, this result again highlights a beneficial impact of fear of COVID-19 on the relationship between religiosity and well-being in late adolescents. Based on the Meaning-Making Model, it seems very likely that among religiously oriented adolescents, concerns and anxiety about the danger of coronavirus initiated meaning-making processes directed at comprehending the complex situation and regaining consistency within the framework of personal beliefs and goals, which led to less negative emotions, and consequently to higher SWB (Park, 2010, 2013). In this sense, fear of COVID-19 tends to buffer the experiences of negative emotions during the pandemic in that motivated young people. This interpretation harmonises with previous studies which consistently showed positive links between religious behaviour and well-being (Krok, 2014; Villani et al., 2019).

Another interpretation may refer to situational meaning; namely, fear of COVID-19 is also likely to influence late adolescents’ situational meaning by shaping their interpretations of personal, stressful events related to the pandemic from a religious perspective. While encountering potentially stressful or threatening events, individuals tend to assign meaning to them in order to reduce tension and anxiety (Park & George, 2013). Fear of COVID-19 can prompt young people to seek in religious beliefs as alternatives for interpreting and appraising events as less threatening. Religious belief systems have been frequently found to provide adolescents with beneficial ways of understanding and reinterpreting difficult situations (Hardy et al., 2019; Schachter & Ben Hur, 2019). In this sense, fear of COVID-19 serves as a trigger that can generate religiously oriented reappraisals that, in turn, enable young people to maintain their mental balance through meaningful and comforting interpretations.

### Limitations of the Study

The present study is not without limitations. First, the participants constituted a sample of predominantly Christian and rather well-educated Polish sample, which limits the possibility of generalising the results to other more secular contexts. Our findings should thus be compared with caution to the results obtained in other, more religiously diversified countries. Second, due to the cross-sectional nature of our study, it does not allow one to draw causal conclusions. Therefore, the mediation effects can only be treated within the hypothesised model, and future research needs to implement a longitudinal design to establish causal relationships among religiosity, meaning-making, and well-being. Third, we did not examine the specific characteristics of meanings made by participants. The multifaceted nature of the COVID-19 pandemic may have created situations in which not all meanings were positive. Even though individuals can successfully find meaning, this process involves the presence of negative feelings, which in turn could interact with fear of COVID-19. Consequently, future research requires other methods beyond reflective data collection that would differentiate both forms of negative emotional experiences.

## Conclusion

In summary, the present study provides justified support that meaning-making and fear of COVID-19 influence the relationship between religiosity and SWB among late adolescents. Specifically, this study demonstrated the importance of examining the moderated mediation effects with meaning-making being a mediator and fear of COVID-19 as a moderator. Additionally, investigating SWB cognitive and affective indices provided deeper insight into the complex and interactive nature of relationships among religiosity and different facets of well-being in young people affected by the coronavirus pandemic. Especially interesting and useful were the indirect effects of meaning-making and fear of COVID-19 that revealed an interplay of cognitive (i.e. beliefs and goals) and emotional (i.e. fear and anxiety) processes in shaping adolescent well-being. Young people who showed both higher levels of insight into themselves and their worldviews as a result of meaning-making and awareness of threats posed by COVID-19 were able to productively use their religious resources to maintain well-being.

## Data Availability

The supplementary material is accessible at the OSF HOME repository, https://osf.io/2wvf8/.
